# Two Simple Methods of Curing Pediculosis Capitis Quickly

**Published:** 1908-01-04

**Authors:** 


### Points in Treatment.

### TWO SIMPLE METHODS OF CURING PEDICULOSIS CAPITIS QUICKLY.

There are many different applications that will destroy lice in the hair; indeed, almost any strong antiseptic will do so. There is, however, a certain amount of danger in using a solution of such a poison as corrosive sublimate, and although a lotion containing the latter or a similar germicide will kill the pediculi, it is as well to use something less dangerous if it will act as surely. The desiderata of the application are:—

1. That it should act with certainty.
2. That it should act with rapidity.
3. That it should be as free as possible from danger to the patient and to others.
4. That it should not be detrimental to the scalp or hair.
5. That it should destroy the vitality of the nits as well as that of the adult pediculi.
6. That it should be as inexpensive as possible.

Pediculosis capitis is mainly an affection of the poor; in the out-patient department of a large hospital it is extremely common; in middle and upper-class practices it is rare; but cases occur even among the rich; the heads of the children of the well-to-do are not infrequently contaminated accidentally, and now and then a lady consults her doctor, and is aghast to find that her head has become infected in some way by the wretched creatures. To remove the pediculi with all speed is what the doctor is asked to do; and the following are two very simple and most efficient methods of doing so:—

### Rectified or Methylated Spirit.

The patient's hair is all thrown forwards over the forehead, and the patient sits or stands with her face looking down into a large basin; the latter should be far enough below the patient's face to allow of the hair hanging free. The choice between methylated and rectified spirit rests upon two points—the former is the cheaper, but leaves its smell longer in the hair. A pint or a pint-and-a-half of the spirit is taken in a jug, and poured slowly but steadily on to the patient's scalp; it is well to begin at the roots of the hair behind, and gradually move the jug up towards the crown, making sure that the spirit reaches every portion of the scalp from nape of neck to forehead, and from ear to ear. There is no need to pour the spirit down the hair itself—it will run down it from the scalp; and the pediculi, which may have been difficult to detect at first, will now be seen scrambling from the roots of the hair along towards the free ends, and scores, if not hundreds of them will spontaneously tumble off into the basin. It takes, perhaps, five minutes to apply the spirit in this way; and then the hair may be combed with a wide-toothed comb, or brushed, to get rid of more of the pediculi. The spirit dries spontaneously, so that the hair may be now roughly done up, and the patient can go to bed, preferably with a towel laid over the pillow. A single application may be sufficient, but if the patient be a lady she will be so anxious to be certain of the cure that she will repeat the process for three consecutive nights. It is not necessary, nor even advisable, that she should do her hair in any but the ordinary way during the day-time. The pediculi will all be gone after the applications, and the nits will all be dead. The shells of the nits, however, are so firmly stuck on to the hair near the roots that they will still be found if looked for; the persistence of the nit-shells must not be taken to mean persistence of the pediculosis; the pediculi are gone and the nits are dead; it only requires that the hair should be carefully brushed and combed for the nits all to come off in time.

There is one disadvantage in the method, that it makes the skin smart at any spot where it has been broken, or where there has been sufficient irritation of the skin by the pediculi to cause an actual dermatitis. The smarting may be sharp, but it is of short duration; and the spirit, if it can be borne, assists in the healing of the irritated skin. A word of warning is necessary in regard to naked lights—no candle-, gas-, or match-flame must be allowed near the hair during the application of the spirit.

The advantages of the method are obvious, particularly its cleanliness and its ease. The hair does not suffer at all, or, at most, loses a little lustre for a time; the lustre can be restored almost at once when the cure is complete, by washing the hair with a little milk, and allowing it to be exposed to the sun for an hour after the milk has been applied.

### Oil of Sassafras.

This has the advantage of not causing so much smarting if there are sore places on the scalp; it has the same advantages as the spirit in regard to simplicity, ease, cheapness, and rapidity; but it has the two-fold disadvantage of being oily and, therefore, less cleanly than spirit, and of requiring the application to be kept on continuously for 24 hours, so that the patient is prevented from going out naturally for one day at least; it is obvious that the hair is being treated when the oil is used, but when the spirit method is employed there is nothing to indicate next day that the individual is receiving any treatment for the head at all.

Sassafras oil is obtained by distillation from the root and root bark of *Sassafras officinale*, a native of North America. The oil is a yellow or reddish-yellow liquid, with a characteristic odour and a warm aromatic taste. It costs about half-a-crown a pound, and this quantity is sufficient for the treatment of a score of children.

The hair should first be brushed and combed as well as may be possible; the brush is then dipped into a saucerful of oil of sassafras, and the hair well brushed again. When all the hair has become moderately oiled in this way it should be plaited and coiled up, and kept so under a close-fitting cap for 24 hours. On combing and brushing the hair after this the dead pediculi will be swept out.

The main drawback to the oil is its smell; though this is a minor evil in comparison with the pediculi themselves. If the spirit treatment can be borne in spite of the temporary smarting mentioned above it will in most cases probably be preferred.

